# Effect of maternal vaccination on infant morbidity in Bangladesh

**DOI:** 10.1186/s12889-024-18486-x

**Published:** 2024-05-02

**Authors:** Shiqiao Zhao, Jing Zhang, Chenxin Zhang, Mark C. Steinhoff, Yanting Zhang, Bin Zhang

**Affiliations:** 1 Department of Environmental Health, Harvard University, Boston, MA USA; 2 Department of Statistics, Miami University, 334B Upham Hall, Oxford, OH 45056 USA; 3 Department of Epidemiology and Population Health, Albert Einstein College of Medicine, Bronx, NY USA; 4 Global Health Center, Cincinnati Children’s Hospital, Cincinnati, OH USA; 5 Division of Biostatistics and Epidemiology, Cincinnati Children’s Hospital Medical Center, 3333 Burnet Ave, Cincinnati, OH 45229 USA; 6 Department of Pediatrics, University of Cincinnati College of Medicine, Cincinnati, OH USA

**Keywords:** Maternal influenza vaccination, Infant morbidity, Risk factor, Randomized trial, Bangladesh

## Abstract

**Background:**

Risk factors of infant mortality in Africa and south Asian countries have been broadly discussed. However, infant morbidity is largely underestimated. We analyzed the data from a randomized vaccine trial in Bangladesh to identify and assess the effect of risk factors on infant morbidity.

**Methods:**

Pregnant women were randomly assigned to receive either inactivated influenza vaccine or pneumococcal polysaccharide vaccine and the infants were randomly assigned to receive 7-valent pneumococcal conjugate vaccine or Hib conjugate vaccine at week 6, 10 and 14. The data were collected from August 2004 through December 2005. Each pair of infant and mother were followed for 24 weeks after birth with weekly visits. Generalized estimating equations (GEE) for repeated measurements and Poisson regression models were used to identify the risk factors and evaluate their effect on the longitudinal incidence and total number of episodes of respiratory illness with fever (RIF), diarrhea disease, ear problem and pneumonia.

**Results:**

A total of 340 pregnant women were randomized with mean age of 25 years. The baseline mother and infant characteristics were similar between two treatment groups. Exclusive breastfeeding and higher paternal education level were common factors associated with lower infant morbidity of RIF (adjusted OR = 0.40 and 0.94 with *p* < 0.01 and *p* = 0.02, respectively), diarrhea disease (adjusted OR = 0.39 and 0.95 with *p* < 0.01 and *p* = 0.04, respectively), and ear problem (adjusted OR = 0.20 and 0.76 with *p* < 0.01 and *p* < 0.01, respectively). Maternal influenza vaccine significantly reduced the incidence of RIF (adjusted OR = 0.54; *p* < 0.01) but not diarrhea disease or ear problem (*p* > 0.05). Female infants had lower incidence of diarrhea disease (adjusted OR = 0.67; *p* = 0.01) and ear problem (adjusted OR = 0.12; *p* = 0.01).

**Conclusions:**

Maternal influenza vaccination, exclusive breastfeeding, female children, and higher paternal education level significantly reduced the infant morbidity within the 24 weeks after birth in Bangladesh.

**Supplementary Information:**

The online version contains supplementary material available at 10.1186/s12889-024-18486-x.

## Abstract (summary)

Maternal influenza vaccination, exclusive breastfeeding, female children, and higher paternal education level were associated with lower infant morbidity and lower number of episodes of respiratory illness with fever (RIF), diarrhea disease, ear problem, but not pneumonia.

**Supplementary Information:**

The online version contains supplementary material available at 10.1186/s12889-024-18486-x.

## Introduction

According to the World Health Organization (WHO), an estimated 5 million children under the age of 5 years died in 2020 [1]. More than 80% of the deaths occurred in sub-Saharan Africa and southern Asia [1]. Child mortality rate in sub-Saharan Africa is the highest in the world with 74 deaths per 1000 live births which is 14 times higher than the risk in Europe and North America [1]. Risk factors of infant mortality in Africa and southern Asian countries have been broadly discussed [2–5]. However, the risk factors of infant’s morbidity is less researched [6]. Influenza virus is one of the leading causes of infants’ and young children’s mortality and morbidity in developing countries [7–9]. A recent randomized trial conducted in rural Nepal showed that maternal influenza vaccination protects mothers and their infants in low resource settings [10]. The authors found that infant influenza incidence risk ratio decreased by about 30% for those whose mothers received maternal influenza vaccination. In addition to vaccination, risk factors of infant morbidity are still not well understood in regions in Africa and Asia countries with limited food and medical resources. Most of the existing studies focused on a specific disease such as influenza, pneumonia [11, 12]. Very few studies considered the risk factors on incidence of multiple illnesses.

Influenza vaccination is recommended by the WHO for pregnant women. France et al. (2006) suggested the maternal influenza vaccination reduced the incidence rate of neonatal respiratory illness [13]. However, maternal vaccination during pregnancy is not standard policy in many low- and middle-income countries [14]. Especially in south Asian countries like Bangladesh, children grow up with limited food availability and increased exposure to infection [15]. The morbidity rate is significantly higher than developed countries.

We analyzed the data from a randomized trial conducted in Bangladesh. Pregnant women were recruited and randomized to receive either inactivated pneumococcal polysaccharide vaccine or influenza vaccine. Their children were randomized to receive 7-valent pneumococcal conjugate vaccine (PCV7) or Hib conjugate vaccine (Hib) at week 6, 10 and 14. We analyzed the infant morbidity of respiratory illness with fever (RIF), diarrhea, ear problem and pneumonia in the first six months of life.

## Method

### Study design

We analyzed the data that were collected in the Mother’s Gift study. The details of the Mother’s Gift project can be found elsewhere [16, 17]. This was a prospective, controlled, double blinded, randomized trial to assess the safety and immunogenicity in pregnant women of pneumococcal vaccine, as well as the clinical effectiveness of influenza vaccine. The study was conducted in Dhaka, Bangladesh from August 2004 through December 2005 and each pair of mother and infant was interviewed weekly until 24 weeks after birth. Healthy pregnant women who met the inclusion criteria were randomly assigned to the treatment group which received inactivated influenza vaccine (Fluarix, GlaxoSmithKline; 2004, South hemisphere formulation) or control group which received 23-valent pneumococcal polysaccharide vaccine (Pneumovax, Merck) with a 1:1 ratio. Pregnant women received the vaccine after they were enrolled and randomized in the study. Infants of each group were randomized to receive either three doses of pneumococcal conjugate vaccine (Prevnar, Wyeth) or Haemophilus influenzae type b conjugate vaccine (Hiberix, GlaxoSmithKline) with a 1:1 ratio. The randomization was generated by a statistician and was stratified by a study site with a block size of four. Written consent forms were provided to all participants.

After birth, all infants were followed for 24 weeks. Enrollment information, socioeconomic status and delivery related information were collected at baseline. The enrollment information included mothers’ demographics and obstetric history. Social economic status included maternal and paternal education, household information, smoking status of household members. And delivery related information included gestational age, mode of delivery (normal/caesarean/episiotomy, etc.), anesthesia given to mother, complication and baby’s birth weight, height, and sex.

During the first 24 weeks after birth, we had weekly visits or phone calls for each family to ask and record the infant’s morbidity information in the last week including respiratory illness with fever (RIF), diarrhea illness, pneumonia, and ear problem which included ear perforation/discharge and otitis media. All mothers were given and taught to use digital thermometers. Infants who were ill were asked to bring to the study site for lab testing and evaluation. We also asked the parents to report the infant’s feeding in the past three days before each visit and options include breast milk only, breast milk and water, breast milk and other milk, breast milk and food, other milk and water, etc. The treatment of the illness was also recorded. We analyzed the data by identifying and evaluating the risk factors of the primary endpoints which included RIF, diarrhea illness, pneumonia, and ear problem. The analysis was done on an intent-to-treat basis.

### Statistical analysis

Descriptive analysis was performed to summarize the characteristics of the variables of interest. Continuous variables were presented as mean ± standard deviation (std) or median with interquartile range (IQR) and were compared between maternal vaccination groups using two sample t-test or Mann-Whitney U test. Categorical variables were presented as counts and percentages and were compared between two groups using chi square test or Fisher’s exact test. Since the primary outcomes of RIF, diarrhea illness, pneumonia, and ear problem could occur repeatedly, univariate and multivariable generalized estimating equations (GEE) for repeated measurements were used to assess the association between illness and the risk factors considering the correlation between multiple observations within the same subject. The risk factors included maternal vaccine, exclusive breastfeeding, cesarean delivery, infant sex and birth weight, maternal weight, paternal education, and smoker in household. We also counted the number of episodes for each infant and used Poisson regression models to evaluate its relationship with the risk factors. When fitting the Poisson models, we used the total number of weeks with exclusive breastfeeding as the predictor. We also included the total number of weeks in follow up as an offset variable to adjust for the total follow up time. For pneumonia and ear problem, since the number of infants who had at least one episode was small, zero-inflated Poisson (ZIP) model was used to allow for frequent zero-valued observations. Odds ratio (OR) and relative risk (RR) and their 95% confidence intervals (CIs) were reported to evaluate the impact of the risk factors. Model selection was based on stepwise criterion which minimized the quasi-information criterion (QIC) for GEE or Akaike information criterion (AIC) for Poisson regression. All analyses were performed using SAS version 9.4 (SAS Institute Inc., Cary, NC). A p-value less than 0.05 was considered statistically significant.

## Result

A total of 340 pregnant women who met the inclusion and exclusion criteria were enrolled and vaccinated in the study between August 2004 and May 2005. Among them, 9 were excluded from the analyses (5 migrated out and 4 stillbirth). As a result, 166 mothers were randomized to the intervention group which received influenza vaccine and 165 were assigned to the control group which received pneumococcal vaccine. Three hundred and thirty-one mothers and their infants were followed and observed from birth through 24 weeks of age. For the 331 pregnant women, the mean age was 25 years old and 45.3% of pregnant women have at least one time of obstetric history (term births, preterm births, abortions, living children); 47.1% of the births were cesarean deliveries. With only 6% low birth weight (< 2.5 kg) and no deaths, most of the infants were healthy. The mean birth weight of babies was 3.1 kg. Mean exclusive breastfeeding time was about 14 weeks. Maternal and paternal education years were 11 and 13 years on average; 42.3% families had at least one person smoking in the household. There was no significant difference in baseline demographics and characteristics in mothers or infants between two vaccination groups (Table 1). Fourteen infants were followed less than 24 weeks and 7 were followed over 30 weeks. All available data were included in the GEE and Poisson regression models.

**Table 1:** Characteristics of mothers, infants and household members by maternal vaccine group

Characteristic	Pneumococcal vaccine (*N* = 165)	Influenza vaccine (*N* = 166)	P
**Mother**			
Maternal age (yr)	24.9 ± 4.2	25.2 ± 4.2	0.57
Maternal weight (kg)	58.4 ± 9.79	57.9 ± 8.58	0.64
Maternal education (yr)	10.9 ± 3.60	11.3 ± 3.25	0.32
Gravida	2 (1, 3)	2 (1, 2)	0.72
Parity	1 (1, 1)	1 (1, 1)	0.62
**Infant**			
Female children	71 (43.0%)	75 (45.2%)	0.69
Birth weight (kg)	3.0 ± 0.07	3.1 ± 0.07	0.10
Exclusive breastfeeding (wk)	14.6 ± 8.36	13.5 ± 8.53	0.24
Cesarean delivery	79 (47.9%)	77 (46.4%)	0.79
**Household Members**			
Paternal education (yr)	12.8 ± 3.33	13.2 ± 2.79	0.27
Smoker in household	75 (45.5%)	66 (39.8%)	0.30

Seventy-nine (48%) infants whose mothers received influenza vaccine experienced at least one RIF during the follow up time with a total of 178 episodes, compared to 110 (66%) infants in the control group experienced 307 episodes. Univariate GEE showed exclusive breastfeeding (OR = 0.41; *p* < 0.01), maternal influenza vaccine (OR = 0.58; *p* < 0.01) and higher paternal education level (OR = 0.94; *p* = 0.02) were significantly associated with reduced incidence of RIF. These factors remained significant in multivariable GEE after model selection with similar ORs (Table 2). When fitting the univariate Poisson regression models, we found that total number of exclusive breastfeeding (RR = 0.99; *p* = 0.03), cesarean delivery (RR = 0.77; *p* < 0.01), maternal influenza vaccine (RR = 0.59; *p* < 0.01) and higher paternal education level (RR = 0.94; *p* < 0.01) significantly reduced the total number of RIF episodes. After adjusting for other covariates, these factors were still significant in the final multivariable model after model selection (Table 2).

**Table 2:** Effect of characteristics on incidence and number of episodes of RIF

Characteristic	GEE				Poisson Model			
	Unadjusted OR (95% CI)	P	Adjusted OR (95% CI)	P	Unadjusted RR (95% CI)	P	Adjusted RR (95% CI)	P
Exclusive breast-feeding*	0.41 (0.31, 0.53)	< 0.01	0.40 (0.30, 0.52)	< 0.01	0.99 (0.98, 1.00)	0.03	0.98 (0.97, 0.99)	< 0.01
Cesarean delivery	0.76 (0.57, 1.00)	0.05	0.79 (0.60,1.04)	0.09	0.77 (0.64, 0.92)	< 0.01	0.80 (0.67, 0.96)	0.02
Female children	0.81 (0.60, 1.01)	0.15			0.83 (0.69, 0.99)	0.04		
Birth weight (kg)	0.90 (0.64, 1.26)	0.54			0.92 (0.76, 1.12)	0.41		
Maternal influenza vaccine	0.58 (0.43, 0.78)	< 0.01	0.54 (0.40,0.72)	< 0.01	0.59 (0.49,0.71)	<0.01	0.59 (0.49,0.71)	<0.01
Maternal weight (kg)	0.99 (0.97, 1.00)	0.10			0.99 (0.98, 1.00)	< 0.01		
Paternal education (yr)	0.94 (0.89, 0.99)	0.02	0.94 (0.89, 0.99)	0.02	0.94 (0.91, 0.96)	< 0.01	0.94 (0.92, 0.97)	< 0.01
Smoker in household	1.15 (0.86, 1.54)	0.36			1.12 (0.94, 1.34)	0.20		

*Exclusive breastfeeding was defined as the total number of weeks with exclusive breastfeeding during follow up in Poisson regression models

Eighty-one (49%) infants in the maternal influenza vaccine group had at least one diarrhea disease during the follow up with a total of 210 episodes, which was less than 91 (55%) infants in the control group with 197 episodes. Multivariable GEE showed exclusive breastfeeding (OR = 0.39; *p* < 0.01), female infant (OR = 0.67; *p* < 0.01) and paternal education (OR = 0.95; p value = 0.04) were significantly associated with reduced incidence of diarrhea disease (Table 3). The same risk factors were also significant in Poisson models which indicates they were also associated with the number of episodes of diarrhea disease with adjusted RR of 0.97, 0.70 and 0.95 with all *p* < 0.01 for exclusive breastfeeding, female infant, and paternal education, respectively. Of note, higher maternal weight was also associated with lower number of weeks with diarrhea disease with adjusted RR of 0.98 and *p* = 0.01.

**Table 3:** Effect of characteristics on incidence and number of episodes of diarrhea disease

Characteristic	GEE				Poisson Model			
	Unadjusted OR (95% CI)	P	Adjusted OR (95% CI)	P	Unadjusted RR (95% CI)	P	Adjusted RR (95% CI)	P
Exclusive breast-feeding*	0.39 (0.29, 0.52)	< 0.01	0.39 (0.29, 0.53)	< 0.01	0.98 (0.97, 0.99)	<0.01	0.97 (0.96, 0.98)	< 0.01
Cesarean delivery	0.96 (0.69, 1.32)	0.79			0.96 (0.79, 1.17)	0.67		
Female children	0.60 (0.43, 0.83)	< 0.01	0.67 (0.48, 0.92)	0.01	0.61 (0.50, 0.75)	< 0.01	0.70 (0.57, 0.87)	< 0.01
Birth weight (kg)	1.05 (0.77, 1.42)	0.76			1.05 (0.85, 1.29)	0.64		
Maternal influenza vaccine	1.05 (0.76, 1.45)	0.76			1.06 (0.87, 1.29)	0.57		
Maternal weight (kg)	0.98 (0.97, 1.00)	0.06			0.98 (0.97, 0.99)	< 0.01	0.98 (0.97, 1.00)	0.01
Paternal education (yr)	0.96 (0.92, 1.00)	0.06	0.95 (0.91, 1.00)	0.04	0.96 (0.93, 0.99)	< 0.01	0.95 (0.93, 0.98)	< 0.01
Smoker in household	1.08 (0.78, 1.48)	0.65			1.05 (0.87, 1.28)	0.61		

*Exclusive breastfeeding was defined as the total number of weeks with exclusive breastfeeding during follow up in Poisson regression models

There were only 9 (5.5%) and 7 (4.2%) infants in the maternal influenza vaccine group who had at least one ear problem and pneumonia with a total of 19 and 12 episodes, respectively. Among the infants in the control group, 12 (7.2%) and 5 (3.0%) had a total of 36 and 11 episodes of ear problem and pneumonia, respectively. Although the number of episodes were small, results from multivariable GEE suggested exclusive breastfeeding (OR = 0.20; *p* < 0.01), female infant (OR = 0.18; *p* < 0.01) and paternal education (OR = 0.76; p value < 0.01) were significantly associated with reduced incidence of ear problem (Table 4). ZIP models were used to evaluate the impact of risk factors on the total number of ear problem episodes to consider the large number of 0s in the outcome. In the final model, female child had lower incidence of ear problem (adjusted OR = 0.23; *p* = 0.04) and higher paternal education reduced the total number of episodes of ear problem (adjusted RR = 0.84; *p* < 0.01) (Table SA1). Also, no significant predictors were found in GEE or ZIP models for pneumonia. The details were not shown here.

**Table 4:** Effect of characteristics on incidence of ear problem

Characteristic	Unadjusted OR (95% CI)	P	Adjusted OR (95% CI)	P
Exclusive breast-feeding*	0.22 (0.08, 0.60)	< 0.01	0.20 (0.07, 0.57)	< 0.01
Cesarean delivery	0.36 (0.13, 1.04)	0.06		
Female children	0.12 (0.03, 0.50)	< 0.01	0.18 (0.04, 0.78)	<0.01
Birth weight	1.13 (0.47, 2.71)	0.79		
Maternal influenza vaccine	0.54 (0.18, 1.60)	0.27		
Maternal weight (kg)	0.94 (0.87, 1.01)	0.11		
Paternal education (yr)	0.77 (0.68, 0.88)	< 0.01	0.76 (0.67, 0.86)	< 0.01
Smoker in household	1.47 (0.45, 4.80)	0.52		

## Discussion

The impact of maternal vaccine on respiratory illness of pregnant women has been discussed elsewhere [18]. However, the impact of maternal vaccines on infants’ and young children’s illness, especially in underdeveloped countries like Bangladesh, is not very clear. The strengths of the Mother’s Gift study include effective randomization of the subjects, a blinded design, and long-term follow-up of outcomes. More details about the design of the study can be found in Zaman et al. (2008). In this paper, we showed that exclusive breastfeeding and higher paternal education level were common factors associated with lower infant morbidity of RIF (adjusted OR of 0.40 and 0.94 with *p* < 0.01 and *p* = 0.02, respectively), diarrhea disease (adjusted OR of 0.39 and 0.95 with *p* < 0.01 and *p* = 0.04, respectively), and ear problem (adjusted OR of 0.20 and 0.76 with *p* < 0.01 and *p* < 0.01, respectively). Pregnant women were randomized to receive either inactivated influenza vaccine or pneumococcal polysaccharide vaccine. Inactivated influenza vaccine provided the protective effect on the RIF and pneumococcal polysaccharide vaccine protected the vaccinator from the pneumonia. We assumed that these maternal vaccinations also provide the protect for the infants on the RIF and pneumonia separately. We showed that maternal influenza vaccine significantly reduced the incidence of RIF (adjusted OR = 0.54; *p* < 0.01) but not diarrhea disease or ear problem (*p* > 0.05). This also confirms the results in the literatures that maternal vaccine and exclusive breastfeeding reduced the infant morbidity rate in populations with limited food and medical resources which are common settings that many infants grow up in Africa and south Asia [19, 20]. Most of the published studies focused on mortality or one specific illness, e.g., influenza, or pneumonia. But very few studies considered multiple illnesses. In this study, in addition to RIF and ear problem, we also analyzed the risk factors of pneumonia and diarrheal disease which are two of the top three causes of child mortality (pneumonia, diarrheal disease and malaria) reported by WHO [21]. Also, our study showed that the female infants had lower incidence of diarrhea disease (adjusted OR = 0.67; *p* = 0.01) and ear problem (adjusted OR = 0.12; *p* = 0.01). It was consistent with previous studies [22, 23]. There is one possible reason that in young infants, immune responses to vaccines were consistently higher or equivalent in girls compared with boys and in general, females typically develop higher antibody responses [24, 25]. We also tried to explore the effect of infants’ vaccination on the outcomes. However, the impact of infants’ vaccination wasn’t significant on the RIF or pneumonia.

Another interesting and important finding is about the impact of parent’s education on infant morbidities. To our best knowledge, almost all published studies used maternal education level as a potential risk factor of infant morbidity. However, we found paternal education level was a more significant predictor. In our dataset, there was no single parent family. Among 340 families, 240 families’ heads were the husbands, and 44 families’ heads were the participants’ father-in-law which means the head of 84% families was a male member. It is well known that the education level is a critical indicator in social economics, e.g., it is highly correlated with income [26–28]. We assessed the univariate correlation between illness and maternal education level and found that it was less significantly or non-significantly correlated with all illness in all models compared to paternal education level (Table SA2). We also replaced paternal education levels with maternal education levels in all multivariable models or included both in the model and then did model selection. In some models, the maternal education was not significant in the multivariable model after adjusting for other covariates. Paternal education level was more significant to stay in all final models. The correlation between maternal education levels and paternal education level was 0.75 in our data which indicates a strong correlation. To avoid multicollinearity, variables with strong correlation may not be included in the same model, it is suggested from our data that paternal education level might be considered as a more important factor in studies considering social economics in similar settings as our study population.

Mother’s Gift project was a prospective, controlled, blinded, randomized trial and weekly data were collected for infants through 6 months of age which made the study unique. Although this was a very well-designed study, there were still some limitations. First, only influenza and clinic visits were laboratory confirmed, most of the surveys were self-reported. Some of the participants missed one or more visits, the information on some of the questions may not be very accurate, e.g., the duration of illness. Second, as most of the longitudinal studies require periodic follow-ups, not all participants followed the exactly scheduled visit time. It was very difficult to match a visit date to a specific week. We set up a general time window for each week, but there were still some special cases that needed to be considered individually. Moreover, we did not consider the impact of maternal side effect, maternal morbidity or infant vaccine on infant morbidity in this study. One limitation of the study is that both vaccines have similar impact on respiratory illness, e.g. RIF and pneumonia. Thus, the effect of the intervention could be underestimated.

We confirmed that the maternal influenza vaccine and breast milk feeding have a protective effect on the infant morbidity rate. Our findings in Bangladesh could not represent all African and southern Asia countries, however, we think maternal influenza vaccination can protect both mothers and their children in counties and areas with reduced food, clean water, and medical resources. It should be given to pregnant women in underdeveloped regions although it has been recommended by WHO.

### Electronic supplementary material

Below is the link to the electronic supplementary material.

Supplementary Material 1

## Data Availability

The data that support the findings of this study are available on request from the corresponding author, BZ, upon reasonable request. The data are not publicly available due to their containing information that could compromise the privacy of research participants.
