# Supplementary material for: Effect of maternal vaccination on infant morbidity in Bangladesh
Source: BMC Public Health. 2024 May 2;24:1213. doi: 10.1186/s12889-024-18486-x (PMC11064391; doi:10.1186/s12889-024-18486-x)
Supplement: Supplementary file 1 — Supplementary Material 1 [file 12889_2024_18486_MOESM1_ESM.docx]

**Supplementary Appendix**

| **SA1. Effect of Characteristics on Ear Problem Using Multivariable ZIP Model** | | | | |
| --- | --- | --- | --- | --- |
|  | Zero inflation parameter estimates | | Count parameter estimates | |
| Characteristic | Unadjusted OR  (95% CI) | P | Adjusted RR  (95% CI) | P |
| Exclusive breast-feeding* | 0.95 (0.89, 1.01) | 0.12 | 1.03 (0.97, 1.10) | 0.30 |
| Female children | 0.23 (0.06, 0.94) | 0.04 | 0.87 (0.25, 3.05) | 0.83 |
| Paternal education (yr) | 0.95 (0.82, 1.09) | 0.45 | 0.84 (0.79, 0.89) | <0.01 |

* Exclusive breastfeeding was defined as the total number of weeks with exclusive breastfeeding during follow up in ZIP models

| **SA2. Effect of Maternal Education on Incidence and Number of Episodes of RIF, Diarrhea Disease and Ear Problem** | | | | | | | | | |
| --- | --- | --- | --- | --- | --- | --- | --- | --- | --- |
|  | **GEE** | | | | **Poisson Model** | | | | |
| **RIF** | | | | | | | | | |
| Characteristic | Unadjusted OR  (95% CI) | P | Adjusted OR  (95% CI) | P | Unadjusted RR  (95% CI) | P | Adjusted RR  (95% CI) | P |  |
| Maternal education (yr) | 0.95 (0.90, 0.99) | 0.03 | 0.95 (0.91, 1.01) | 0.05 | 0.95 (0.93, 0.97) | <0.01 | 0.96 (0.93, 0.98) | <0.01 |  |
| **Diarrhea Disease** | | | | | | | | |  |
| Maternal education (yr) | 0.97 (0.93, 1.02) | 0.24 | 0.97 (0.93, 1.02) | 0.21 | 0.97 (0.95, 1.01) | 0.07 | 0.99 (0.96, 1.02) | 0.39 |  |
| **Ear Problem** | | | | | | | | |  |
| Maternal education (yr) | 0.81 (0.68, 0.97) | 0.02 | 0.80 (0.67, 0.96) | 0.02 | 0.80 (0.75, 0.85) | <0.01 | 0.83 (0.78, 0.89) | <0.01 |  |
